# Effect of Nitric Acid Concentrations on Synthesis and Stability of Maghemite Nanoparticles Suspension

**DOI:** 10.1155/2014/589479

**Published:** 2014-05-18

**Authors:** Irwan Nurdin, Mohd Rafie Johan, Iskandar Idris Yaacob, Bee Chin Ang

**Affiliations:** ^1^Nanomaterials Engineering Research Group, Advanced Materials Research Laboratory, Department of Mechanical Engineering, Faculty of Engineering, University of Malaya, 50603 Kuala Lumpur, Malaysia; ^2^Department of Chemical Engineering, Lhokseumawe State Polytechnic, Lhokseumawe 24301, Indonesia; ^3^Department of Manufacturing and Materials Engineering, Kulliyyah of Engineering, International Islamic University Malaysia, 50728 Kuala Lumpur, Malaysia

## Abstract

Maghemite (*γ*-Fe_2_O_3_) nanoparticles have been synthesized using a chemical coprecipitation method at different nitric acid concentrations as an oxidizing agent. Characterization of all samples performed by several techniques including X-ray diffraction (XRD), transmission electron microscopy (TEM), alternating gradient magnetometry (AGM), thermogravimetric analysis (TGA), dynamic light scattering (DLS), and zeta potential. The XRD patterns confirmed that the particles were maghemite. The crystallite size of all samples decreases with the increasing concentration of nitric acid. TEM observation showed that the particles have spherical morphology with narrow particle size distribution. The particles showed superparamagnetic behavior with decreased magnetization values at the increasing concentration of nitric acid. TGA measurement showed that the stability temperature decreases with the increasing concentration of nitric acid. DLS measurement showed that the hydrodynamic particle sizes decrease with the increasing concentration of nitric acid. Zeta potential values show a decrease with the increasing concentration of nitric acid. The increasing concentration of nitric acid in synthesis of maghemite nanoparticles produced smaller size particles, lower magnetization, better thermal stability, and more stable maghemite nanoparticles suspension.

## 1. Introduction

Due to their unique characteristics, magnetic nanoparticles have received much attention in recent year especially in various fields of application including electronic, mechanical engineering, aerospace, and environmental and bioengineering [1–4]. Iron oxide nanoparticles particularly magnetite (Fe_3_O_4_) and maghemite (*γ*-Fe_2_O_3_) are promising magnetic materials that are intensively explored due to their unique magnetic properties.

Suspended maghemite nanoparticles in solvent creates new class of liquids called “magnetic fluids.” The uniqueness of these smart materials is because of their superparamagnetic property. The flow and energy transport of magnetic fluids can be controlled using external magnetic fields. Therefore, the magnetic fluids can be used effectively in thermal engineering applications [5].

Recently, many attempts have been carried out to synthesize stable magnetic nanoparticles suspension in various methods in order to achieve proper control of particle size, shape, crystallinity, and the magnetic properties [6]. The synthesis of magnetic fluids consists of two main steps: preparation of nanosize magnetic particles and stabilization of magnetic nanoparticles in various liquids. Various methods are available for synthesis of magnetic nanoparticles such as coprecipitation [7, 8], sol-gel synthesis [9], and microemulsion [10]. The most common is the coprecipitation method [7, 8, 11]. This method is reproducible, simple, and cheap and it gives high yield result.

Although many attempts have been made towards the synthesis of stable magnetic nanoparticles suspension, it still presents a big challenge. The most important parameter in the preparation of this materials is the stability of the magnetic nanoparticles suspension.

In this paper, maghemite nanoparticles were synthesized by chemical coprecipitation method at various nitric acid concentrations and characterized by a variety of analytical techniques. The stability of maghemite nanoparticles suspension was analyzed by monitoring their particle size distribution using dynamic light scattering (DLS).

## 2. Materials and Methods

The chemical reagents used in this experiment were ferric chloride hexahydrate (FeCl_3_·6H_2_O), ferrous chloride tetrahydrate (FeCl_2_·4H_2_O), and ferric nitrate nonahydrate (Fe(NO_3_)_3_·9H_2_O). These chemicals were obtained from Sigma-Aldrich. Ammonium hydroxide (NH_4_OH) 28%, nitric acid (HNO_3_) 65%, and hydrochloric acid (HCl) 37% were purchased from Merck Chemical. Deionized water with resistivity of around 15 MΩ/cm was used throughout the experiment. All reagents were analytical grade and were used as received without further purification.

Ferric chloride and ferrous chloride solutions with molar ratio 2 : 1 were mixed together. Then, ammonium hydroxide solution was added into the solution with vigorous stirring for 20 minutes at room temperature. A black precipitate formed instantaneously and was separated from the solution and washed several times by stirring for 5 minutes in deionized water. The precipitate was then stirred for 10 minutes in various nitric acid concentrations (i.e., 2 M, 4 M, 6 M, 8 M, and 10 M). The precipitate was separated and washed several times and then oxidized to maghemite at 90°C for 30 minutes using ferric nitrate solution. Brown precipitate was isolated from solution and then washed and peptized thoroughly with deionized water. The chemical reaction of the precipitation process is given below:

(1) $$\begin{array}{c} 2\text{Fe}{\text{Cl}}_{3}+\text{Fe}{\text{Cl}}_{2}+8\text{N}{\text{H}}_{4}\text{OH}⟶{\text{Fe}}_{3}{\text{O}}_{4}+4{\text{H}}_{2}\text{O}+8\text{N}{\text{H}}_{4}\text{Cl} \end{array}$$

(2) $$\begin{array}{c} 2{\text{Fe}}_{3}{\text{O}}_{4}+\text{HN}{\text{O}}_{3}⟶\gamma \text{-}3{\text{Fe}}_{2}{\text{O}}_{3}+\text{HN}{\text{O}}_{2} \end{array}$$

X-ray powder diffraction (XRD) measurements were carried out with a Philips X'Pert MPD X-Ray Diffractometer, using copper source (*λ* = 1.54056 Å) with a scan range of 20–80° 2*θ* angle at a step of 0.05° and a count time of 5 s at each step. The morphology and physical size of the particles were observed using transmission electron microscopy (TEM). The images were taken using a Leo LIBRA transmission electron microscope operated at 120 kV. The magnetic property of the maghemite was measured by an alternating gradient magnetometer (MicroMag, model 2900) with applied fields of ±10 kOe at room temperature. Thermal gravimetry analysis (TGA) was performed to investigate the effect of the heat treatment on the thermal behavior and physical properties of the samples. TGA analysis was performed from ambient temperature to 1000°C with a heating rate of 10°C/min. The analysis was conducted in air atmosphere. The hydrodynamic diameter and zeta potential of the nanoparticles suspension were determined by dynamic light scattering (DLS) using a Malvern Zetasizer 3000 HS at 25°C.

## 3. Results and Discussion

**Figure 1:**
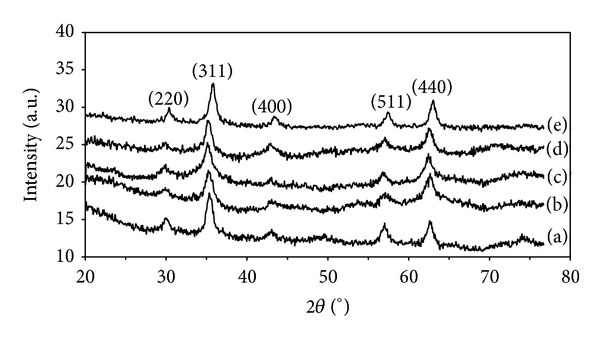
XRD patterns of maghemite nanoparticles for samples: (a) MNA2, (b) MNA4, (c) MNA6, (d) MNA8, and (e) MNA10.

The XRD patterns of all samples are shown in Figure 1. The patterns show well defined peaks which clearly indicate the crystallinity of the samples. The reflection peaks in the pattern were indexed to face center cubic (fcc) phase with lattice parameters (a) 8.375 ± 0.018, 8.375 ± 0.015, 8.368 ± 0.011, 8.356 ± 0.011, and 8.334 ± 0.005 Å for MNA2, MNA4, MNA6, MNA8, and MNA10 samples, respectively. This is in good agreement with the bulk lattice parameter of maghemite (*a* = 8.3474 Å) [12]. The crystallite size of the nanoparticles was calculated from the XRD line broadening using Scherrer's equation as listed in Table 1. It is shown that the crystallite size of nanoparticles is gradually reduced if the concentration of nitric acid is increased.

**Figure 2:**
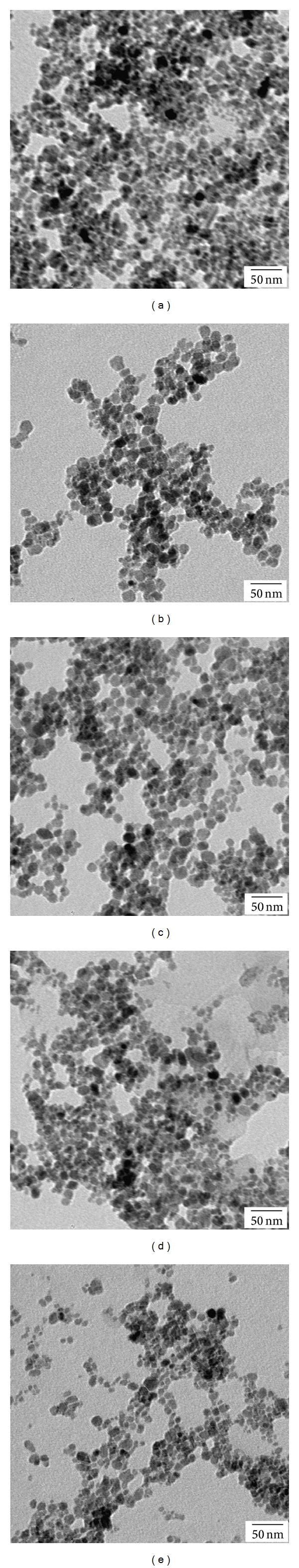
TEM images of maghemite nanoparticles for samples: (a) MNA2, (b) MNA4, (c) MNA6, (d) MNA8, and (e) MNA10.

**Figure 3:**
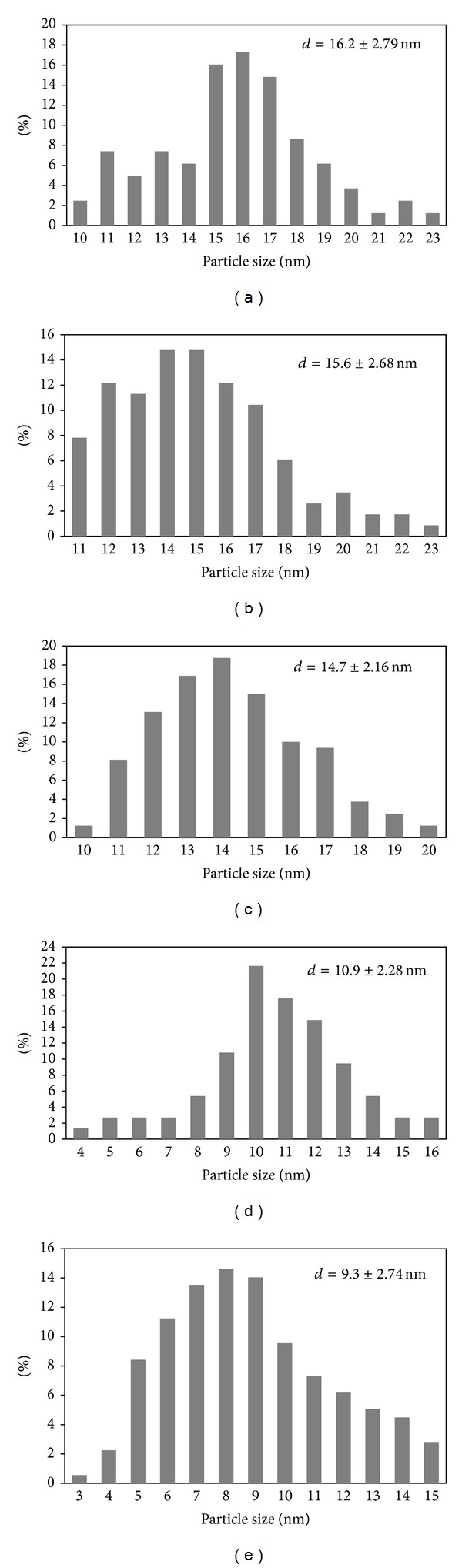
Particle size distribution of maghemite nanoparticles for samples: (a) MNA2, (b) MNA4, (c) MNA6, (d) MNA8, and (e) MNA10.

The shape and particle size distribution of maghemite nanoparticles were examined by transmission electron microscopy (TEM) as shown in Figures 2 and 3. It is clearly observed that the maghemite particles have spherical shape. The sizes of the particles were measured from about 100 particles and listed in Table 1. There are a few larger “particles” which are found to be aggregates, which may be due to long-range magnetic dipole-dipole interaction between the particles. This average physical size is in a good agreement with the crystallite size obtained from XRD measurement indicating that the particles are largely monocrystals.  Figure 3 shows the particles size distribution of maghemite nanoparticles. It is clearly observed that the particles show normal size distribution.

**Figure 4:**
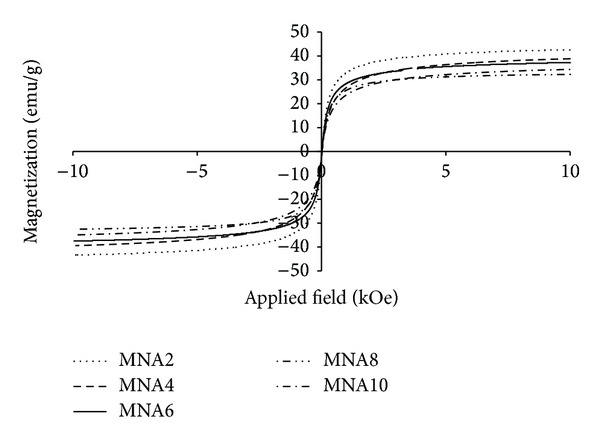
Magnetization curves of maghemite nanoparticles for all samples.

The magnetization curve of all samples is shown in Figure 4. It is clear that the curves do not exhibit hysteresis and passes through the origin, which indicates that the samples are superparamagnetic. The saturation magnetization values of maghemite nanoparticles at room temperature for all samples are tabulated in Table 1. These values are lower than that of bulk maghemite (74 emu/g) due to the fact that the crystallite size of maghemite particles is in nanosize range. This phenomenon is usually observed in nanoparticles interacting systems. Such a reduction of maximum magnetization can be ascribed to surface effects arising from broken symmetry and reduced coordination of atoms lying at the surface of maghemite nanoparticles and also to a high degree of interparticle interactions [13].

**Figure 5:**
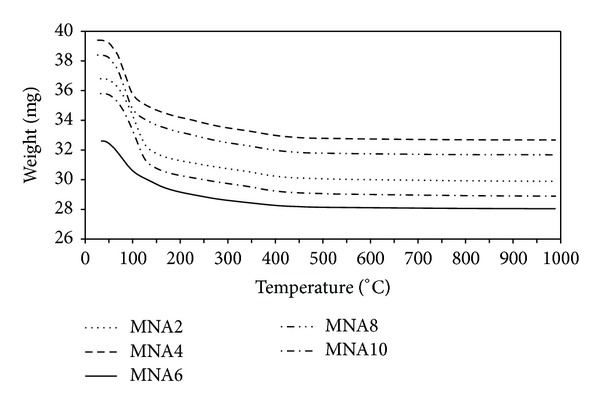
TGA thermograms of maghemite nanoparticles for all samples.

TGA curves of the maghemite nanoparticles at different nitric acid concentrations are shown in Figure 5. It can be seen that the curves exhibit similar weight loss behavior and display two weight losses steps. The initial weight loss starts from the ambient temperature to 140°C and the final weight loss is within the temperature range of 140 to 400°C. The initial weight loss is associated with the evaporation of absorbed water and crystalline water from the sample. The final weight loss might be attributed to the volatilization of the remainder bonding water in the sample which will evaporate at water critical temperature of 374°C. No further significant weight loss or gain is found in the temperature range of 400 to 1000°C, indicating crystalline of maghemite has been formed completely. The temperature stability (*T*_*s*_) for all samples when maghemite is completely formed is presented in Table 1. It can be seen that the temperature stability decreases with increasing concentration of nitric acid. This indicates that sample with the most concentrated nitric acid is stabilized earlier than other samples.

**Figure 6:**
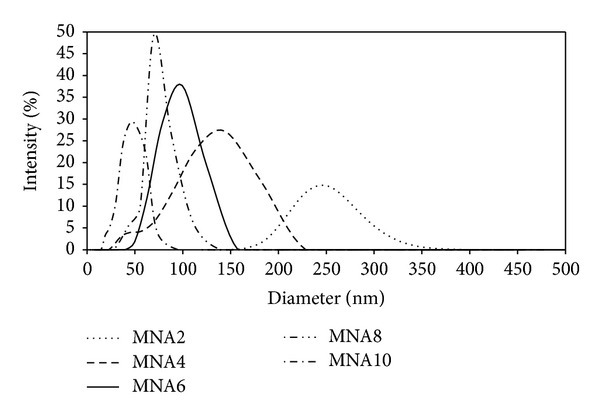
DLS measurement of maghemite nanoparticles for all samples.

The particle size distributions of maghemite nanoparticles suspension obtained from dynamic light scattering (DLS) measurement are shown in Figure 6. It is presented that the intensity averaged particle size of maghemite nanoparticles for all samples. Their values are listed in Table 1, which indicate that the maghemite nanoparticles diameter reduce with increasing concentration of nitric acid. High concentration of nitric acid will suppress double layer of ions around the particles, enhancing the diffusion speed and resulting in smaller hydrodynamic diameter. It is also shown that the particle sizes obtained are larger than the TEM results due to the hydrodynamic diameter of particles and their surrounding solvent layers.

**Figure 7:**
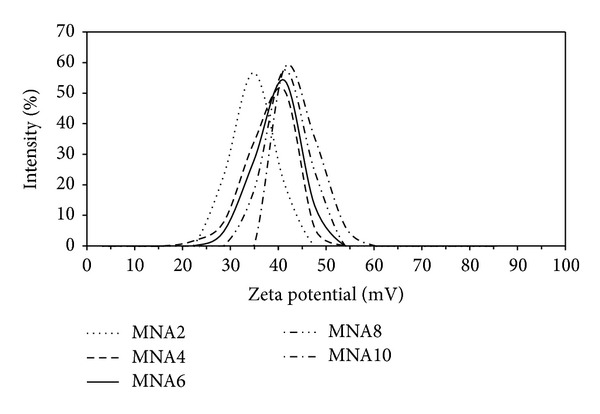
Zeta potential of all samples.

The stability of suspension is related to its electrokinetics properties. Therefore the study of electrophoretic behavior through measurement of zeta potential becomes important for understanding the stability of suspension [14]. It is recognized that nanoparticles suspension becomes stable with a zeta potential value higher than ± 30 mV. The zeta potential of maghemite nanoparticles suspension is shown in Figure 7. The values of zeta potentials are listed in Table 1. These values indicate that the maghemite nanoparticles suspension is stable. Increasing concentration of nitric acid will compress the thickness of double layer, hence increasing the particle charge. As a result, it will increase the zeta potential value.

## 4. Conclusion

Stable maghemite nanoparticles suspension has been successfully synthesized by coprecipitation method at various concentrations of nitric acid.

The patterns obtained from XRD show well defined peaks which clearly indicate that the samples are crystalline. They also reveal that the particles are confirmed maghemite. TEM observations and image analysis show that the maghemite nanoparticles have spherical morphology and small size particles. Magnetization curves show that maghemite nanoparticles exhibit superparamagnetic behavior. The particles show good thermal stability during thermogravimetry analysis.

The increasing concentration of nitric acid in synthesis of maghemite nanoparticles will produce smaller size, lower magnetization, better thermal properties, and more stable maghemite nanoparticles.

## Figures and Tables

**Table 1:** Particles size, magnetic property, zeta potential, and temperature stability (T_S_) of maghemite nanoparticles.

Samples	XRD (nm)	TEM (nm)	AGM (emu/g)	DLS (nm)	Zeta potential (mV)	Temperature stability (°C)
MNA2	14.2 ± 1.20	16.2 ± 2.79	42.4	247.7	36	525
MNA4	13.9 ± 1.15	15.6 ± 2.68	38.6	118.1	37.6	485
MNA6	12.8 ± 0.80	14.7 ± 2.16	36.6	93.1	39.7	475
MNA8	11.4 ± 0.80	10.9 ± 2.28	34.2	73.6	41.7	460
MNA10	10.6 ± 0.81	9.3 ± 2.74	32.1	45.3	44.6	450
